# Mixed neuroendocrine–non-neuroendocrine neoplasms of the gallbladder: a case report

**DOI:** 10.1186/s40792-021-01152-4

**Published:** 2021-03-17

**Authors:** Tatsuki Ishikawa, Katsunori Nakano, Masafumi Osaka, Kenichi Aratani, Kadotani Yayoi, Kiyokazu Akioka, Kuniyuki Tsuchiya, Yohei Hosokawa

**Affiliations:** 1 Department of Surgery, Omihachiman Community Medical Center, 1379 Tsuchidacho, Omihachimanshi, Shiga 523-0082 Japan; 2 Department of Pathology, Omihachiman Community Medical Center, 1379 Tsuchidacho, Omihachimanshi, Shiga 523-0082 Japan

**Keywords:** Mixed neuroendocrine–non-neuroendocrine neoplasms, Mixed adenoneuroendocrine carcinoma, Gallbladder cancer

## Abstract

**Background:**

Primary neuroendocrine tumors of the gallbladder (GB-NETs) are rare, accounting for 0.5% of all NETs and 2.1% of all gallbladder cancers. Among GB-NETs, mixed neuroendocrine–non-neuroendocrine neoplasms of the gallbladder (GB-MiNENs) are extremely rare.

**Case presentation:**

We present the case of a 66-year-old woman who was referred to us for the management of a gallbladder tumor (incidentally found during abdominal ultrasonography indicated for gallbladder stones). The patient had no history of abdominal pain or fever, and the findings on a physical examination were unremarkable. Blood tests showed normal levels of tumor markers. Imaging studies revealed a mass of approximately 10 mm in diameter (with no invasion of the gallbladder bed) located at the fundus of the gallbladder. A gallbladder cancer was suspected. Therefore, an open whole-layer cholecystectomy with regional lymph nodes dissection was performed. The postoperative course was uneventful, and she was discharged on postoperative day 6. Pathological findings showed GB-MiNENs with invasion of the subserosal layer and no lymph node invasion (classified T2aN0M0 pStage IIA according to the Union for International Cancer Control, 8th edition staging system). Analysis of the neuroendocrine markers revealed positive chromogranin A and synaptophysin, and a Ki-67 index above 95%. Fourteen months after the operation, a local recurrence was detected, and she was referred to another hospital for chemotherapy.

**Conclusions:**

GB-MiNENs are extremely aggressive tumors despite their tumor size. Optimal therapy should be chosen for each patient.

## Background

Primary neuroendocrine tumors of the gallbladder (GB-NETs) are rare, accounting for 0.5% of all NETs and 2.1% of all gallbladder caners. Among GB-NETs, mixed neuroendocrine–non-neuroendocrine neoplasms of the gallbladder (GB-MiNENs) are extremely rare.

## Case presentation

**Fig.1:**
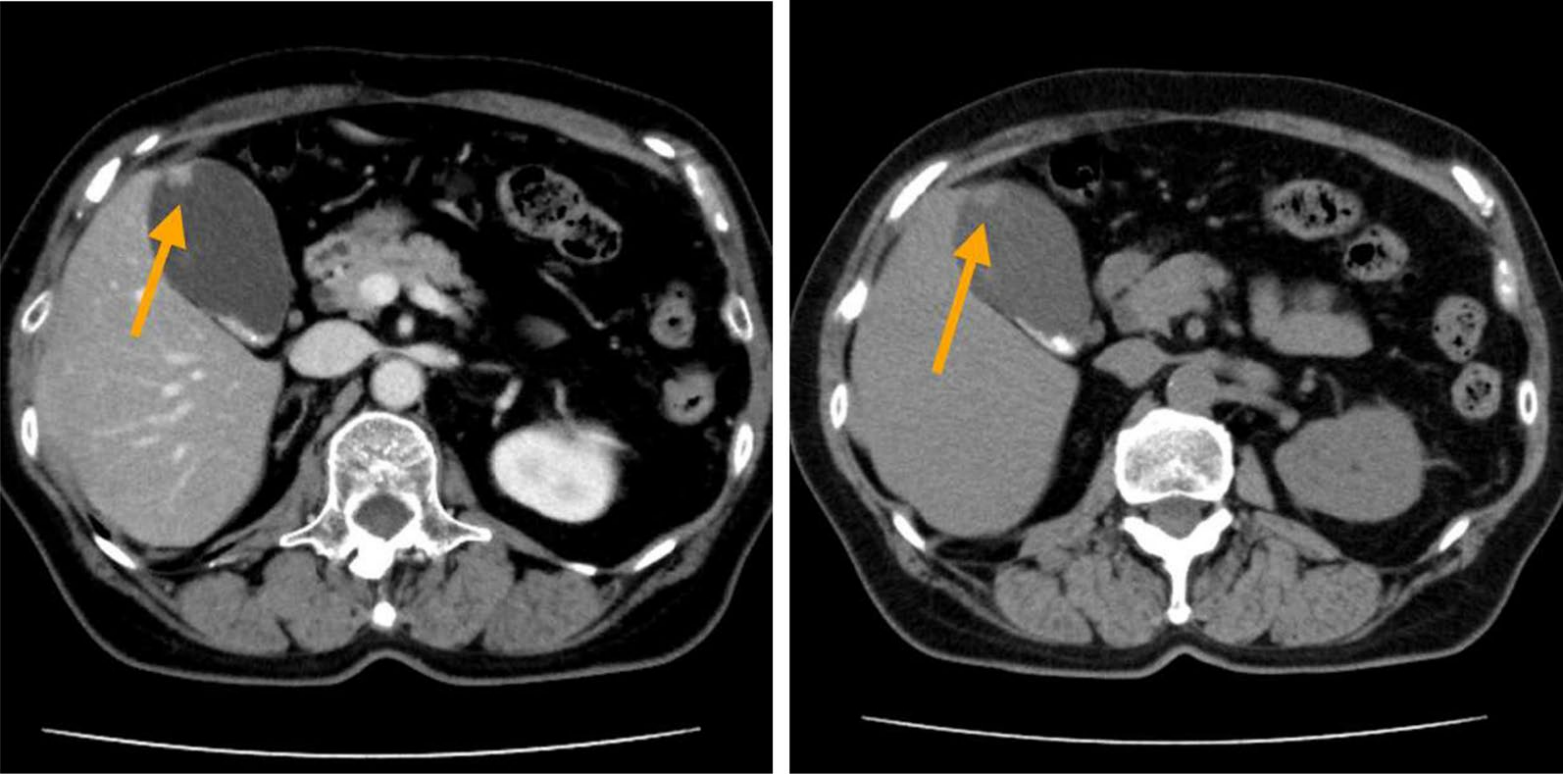
Abdominal computed tomography showed slightly enhanced tumor in the fundus of gallbladder (arrow)

**Fig.2:**
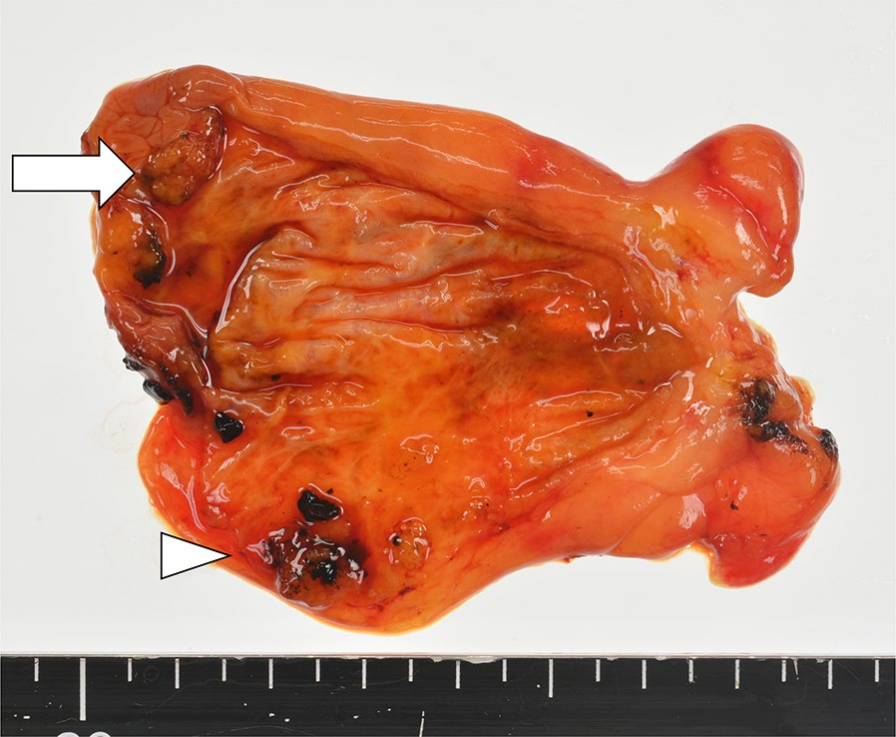
Macroscopic findings showed papillary tumor in the fundus of gallbladder (arrow). Some gallstones existed within the gallbladder (arrowhead)

We present the case of a 66-year-old woman referred for management of a gallbladder tumor, which was incidentally found during an abdominal ultrasonography indicated for gallbladder stones. The patient had no history of abdominal pain or fever, and the findings on physical examination were unremarkable. The blood tests showed normal levels of tumor markers (carcinoembryonic antigen = 2.7 ng/mL, carbohydrate antigen 19–9 = 5.4 U/mL). Contrast-enhanced computed tomography (CT) showed wide-based mass of approximately 10 mm in diameter contrasted in arterial phase at the fundus of the gallbladder. MRI showed a nodule in the fundus of gallbladder with high signal in diffusion-weighted imaging and low in apparent diffusion coefficient. Since there was no Rokitansky–Aschoff sinus in the nodule, the possibility of gallbladder adenomyomatosis was considered low. The tumor was limited within gallbladder; it was hard to predict precise depth of tumor even by radiologist (Fig. 1). A gallbladder cancer was suspected. Therefore, an open whole-layer cholecystectomy with regional lymph nodes dissection (#12c, 12b, 12p, 12a) was performed. The resected specimen showed a nodular-expanding tumor, located at the fundus of the gallbladder, measuring 10 mm in diameter (Fig. 2). The postoperative course was uneventful, and she was discharged on the postoperative day 6.

**Fig.3:**
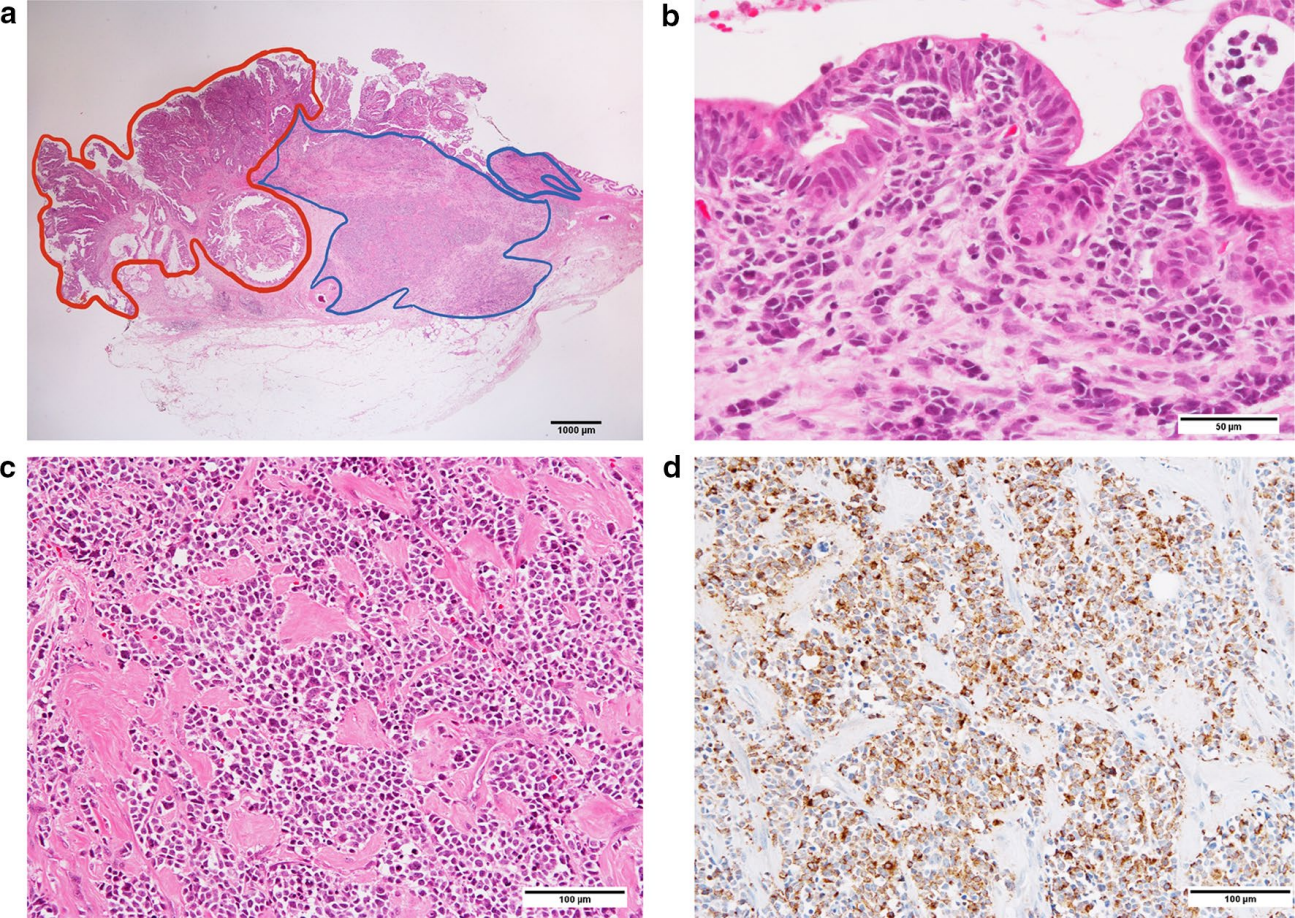
Histological findings of gallbladder tumor. **a** Hematoxylin–eosin staining (× 4). Adenocarcinoma component was located on the surface of tumor (red area), whereas neuroendocrine component in the deeper subserosal layer (blue area). **b** Hematoxylin–eosin staining (× 40). Papillary and tubular adenocarcinoma. **c** Hematoxylin–eosin staining (× 20). Neuroendocrine tumor cells arranged in nests. **d** Immunohistochemical staining for chromogranin A (× 20). Immunohistochemical staining revealed positive expression of synaptophysin

Pathological findings revealed both adenocarcinoma and neuroendocrine components in the tumor. In immunostaining, the neuroendocrine component (which spanned from the submucosal to the subserosal layers) was positive for chromogranin A, synaptophysin, and CD56 (Fig. 3). The Ki-67 index was above 95%, and the neuroendocrine component was considered equivalent to large-cell neuroendocrine carcinoma (LCNEC).

While there was venous infiltration in the adenocarcinoma area, there was no vascular involvement in the neuroendocrine area.

The exocrine (adenocarcinoma) and neuroendocrine components were each present in more than 30% of the tumor mass. Therefore, the tumor was diagnosed as a GB-MiNENs with invasion of the subserosal layer and with no lymph node invasion classified T2aN0M0 pStage IIA (according to the Union for International Cancer Control 8th edition staging system).

After the operation, she received oral adjuvant chemotherapy (tegafur/gimeracil/oteracil potassium combination) for 5 months. She later discontinued the chemotherapy due to loss of appetite and diarrhea. However, 14 months after the operation, a local recurrence was detected. She was referred to another hospital for chemotherapy.

## Discussion

According to the 2019 WHO Classification of Tumors of the Digestive System, MiNENs are defined as tumors consisting of two components, a non-neuroendocrine tumor, and a neuroendocrine tumor, each of which host at least 30% of the total cellular population on pathological examination [1]. English expert literature reports 2 case reports about GB-MiNENs (Table1) [2, 3]. Primary neuroendocrine tumors of the gallbladder (GB-NETs) are rare, accounting for 0.5% of all NETs and 2.1% of all gallbladder cancers [4]. The origin of GB-NETs is uncertain because there are no neuroendocrine cells in the gallbladder. The metaplasia observed in the gallbladder mucosa (especially during chronic inflammation) is similar to that observed in the stomach. It includes two cell metaplasia types: the pyloric or gastric type, and the intestinal type. Gallbladder cancer is believed to originate from atypical hyperplasia of the epithelium [5]. A study reported that neuroendocrine cells originate from stem cells, which have deviated from their original differentiation pattern during the process of metaplasm. This is most common with intestinal-type metaplasia [6]. Cholecystolithiasis and cholecystitis were also found in most cases of GB-NETs, suggesting that GB-NETs might be related to chronic inflammation of the gallbladder. In our case, there was no pathological evidence of chronic inflammation in the gallbladder wall, though the presence of gallstones in the resected specimen might have implied chronic inflammation.

**Table1:** Recent reports of GB-MiNENs

Case no.	Gender/age	Chief complaint	Tumor location/size (cm)	Components	Liver invasion	Metastasis	Treatment	Follow-up (months)	Reference
1	F/56	RUQ pain, weight loss	Gallbladder to liver area/17	AC (35%), SCNEC (65%)	Present	None	Chol, Phx, Chem	Alive (13)	[2]
2	F/62	RUQ pain	Fundus/5	AC (10%), SCC (60%), NEC (30%)	Absent	Umbilicus	Chol, Phx, Chem	Alive (14)	[3]
3	F/66	None	Fundus/1	AC (55%), LCNEC (45%)	Absent	none	Chol, Chem	Alive (14)	Our case

*Chem* chemotherapy, *Chol* cholecystectomy, *LCNEC* large-cell neuroendocrine carcinoma, *Phx* partial hepatectomy, *RUQ* right upper quadrant; *SCC* squamous cell carcinoma; *SCNEC* small-cell neuroendocrine carcinoma

Studies have shown that in most MiNEN, the adenocarcinoma component is located on the mucosal surface, meanwhile the neuroendocrine component invades the deeper areas [7]. In our case, the well-differentiated adenocarcinoma mainly existed between the mucosal and the muscular layers, while the neuroendocrine component was located between the muscular and subserosal layers.

Just as in other gallbladder tumors, no specific symptoms and changes in image findings occur in GB-NET, making its diagnosis difficult. The most common presenting symptom is vague abdominal pain or discomfort and the GB-NET is usually associated with cholelithiasis [8]. A study showed that only 3.3–3.7% of patients with GB-NETs have the carcinoid syndrome [9]. In our case, the patient had no symptoms (including hormone-related symptoms). Like in other gallbladder carcinomas, most GB-NETs are asymptomatic and can readily invade the adjacent liver parenchyma and cause late biliary obstruction. This makes early detection difficult.

Imaging studies like ultrasound, computed tomography, magnetic resonance imaging, positron emission tomography–CT, and somatostatin receptor scintigraphy, reveals useful information about GB-NETs. However, the imaging findings of GB-NETs are non-specific and it is extremely difficult use imaging findings to differentiate between GB-NETs and gallbladder adenocarcinoma. While neuroendocrine tumor is hypervascular tumor strongly enhanced on CT, neuroendocrine carcinoma (NEC) is hypovascular tumor like adenocarcinoma. It is difficult to differentiate NEC from adenocarcinoma. Iwamoto et al. reported that since in the case of NEC of the gallbladder, the progression is more rapid than in the case of gallbladder cancer, and the pathology is less fibrosis and more necrotic changes, tumor showed images with poor contrast effect on late phase of contrast enhanced CT [10]. Murawaki et al. reported diffusion-weighted image (DWI) in MRI was useful for classifying NEC and adenocarcinoma components, because NEC has a higher proliferative capacity than adenocarcinoma, resulting in higher cell density and lower diffusing capacity in NEC [11]. EUS is useful for differential diagnosis and prediction of the depth diagnosis of gallbladder cancer. In this case, the tumor was located in the fundus, EUS was not performed. The confirmatory diagnosis can only be made by pathological examination. If curative resection is possible, surgery is an option of treatment of NEC. However, there is no consensus about the margins of the resection. Based on the stage of the tumor, various procedures ranging from simple cholecystectomy to radical liver resection with lymphadenectomy and resection of the hepatoduodenal ligament could be performed. In this case, cholecystectomy was performed as diagnostic treatment. For patients with poorly differentiated unresectable NEC, a chemotherapy protocol with cisplatin, carboplatin, and etoposide is usually recommended as primary treatment. Iwasa et al., (2010) reported that cisplatin and etoposide combination had a marginal antitumor activity and a relatively severe toxicity when used as first-line chemotherapy for poorly differentiated hepatobiliary or pancreatic NECs (when compared with extrapulmonary poorly differentiated NECs treated with the same regimen) [12]. Terashima et al., (2012) reported that NEC of hepatobiliary and pancreatic cancer are more resistant to chemotherapy than small-cell lung cancer or primary gastrointestinal cancer, and have a poor prognosis [13]. Randomized phase III study of etoposide plus cisplatin combination therapy versus irinotecan plus cisplatin combination therapy in advanced neuroendocrine carcinoma of the digestive system by Japan Clinical Oncology Group is undergoing now and the results are awaited. Like other gallbladder cancers, GB-NETs have a poor prognosis. Duffy et al., (2008) reported that the median survival time of patients with gallbladder NEC was 9.8 months, which is lower than that of patients with gallbladder carcinoma [14]. Reed et al. reported by using National Cancer Database that patients with primary GB-NETs were 2.3% of all gallbladder tumors. Nearly half of the patients presented with stage IV disease, the median OS for this cohort was 25 months. They also reported that older age, large-cell histology, positive surgical margins were independently associated with decreased survival [15].

In this patient, a local recurrence was detected 14 months after the operation, prompting referral to another hospital for chemotherapy.

## Conclusions

We encountered GB-MiNENs at an early stage, and because they are asymptomatic at this stage, it was difficult to detect them when they were still resectable. GB-NETs are malignant tumors that still have a poor prognosis. More cases need to be studied to help establish a standardized optimal treatment.

## Data Availability

This case report has no dataset.

## Abbreviations

GB-NETs: Neuroendocrine tumors of the gallbladder
MiNENs: Mixed neuroendocrine–non-neuroendocrine neoplasms
CT: Computed tomography
MRI: Magnetic resonance imaging
LCNEC: Large-cell neuroendocrine carcinoma
NEC: Neuroendocrine carcinoma
